# Intake of divalent copper and nickel onto natural zeolite from aqueous solutions: a study in mono- and dicomponent systems

**DOI:** 10.55730/1300-0527.3413

**Published:** 2022-01-03

**Authors:** Burak TEKİN, Ünsal AÇIKEL

**Affiliations:** 1 Department of Chemical Engineering, 19 Mayıs University, Samsun, Turkey; 2 Department of Chemical Engineering, Cumhuriyet University, Sivas, Turkey

**Keywords:** Adsorption, Natural zeolite, mono- and dicomponent adsorption, Cu (II) and Ni (II) contaminants

## Abstract

In this study, the noncompetitive and competitive adsorption process of copper (II) and nickel (II) ions on the natural zeolite were examined in simulated wastewater in a batch system with respect to concentration, pH and temperature. Optimum pH values were found as 5,0 for adsorption of copper and nickel ions on the zeolite. The effect of initial concentration and ambient temperature on the yield of adsorption was examined at this pH value. The equilibrium adsorption data of Cu (II) and Ni (II) onto the zeolite were analyzed by the Langmuir’s and Freundlich’s isotherms, and the experimental metal uptake data fitted well with both isotherm models. In case of the presence of simultaneous multimetal ions in the aqueous phase, the adsorption capacity of the adsorbent is slightly low probably due to the competitive uptake of each metal ion by the adsorbent. For the metal sorption system, the negative Gibbs free energy values show the applicability and spontaneous nature of the metal uptake treatment by the zeolite. The activation energy and enthalpy change of divalent cation adsorption demonstrate that the intake of Cu (II) and Ni (II) onto the zeolite involves not only a chemical adsorption process but also a physical adsorption process.

## 1. Introduction

Apart from exponentially growing the world population, the industrial and technological revolutions have put mankind into another phase that requires more labor force. Today, it is not a secret that this new stage, unfortunately, has ended up with the water- and air-pollution [1, 2]. The former is a serious threat to be urgently prevented for all the living creatures to survive. The water contamination comes into existence when the effluents dumped from some Industry factories, hospitals, households, farms and any other structures contain some harmful microorganisms or toxic chemicals in higher concentrations than being accepted. The high metal ion concentration in wastewaters not only jeopardizes the health of the aquatic creatures but wreaks havoc on Natural habitats, as well [3, 4]. Today, many water-purification processes have been built up and operated either at a small-scale in an academic realm or at a large-scale in private sector in an attempt to minimize the heavy metal concentration in the contaminated waters.

Heavy metal ions, including copper, nickel, arsenic, cadmium and others, in various concentrations, have been evacuated into the aquatic or terrestrial habitats by anthropogenic activities, such as mining operations, metal plating, and other industrial activities [5, 6]. Herein, it is noted that the term “heavy metal” ascribes to the situations where alkaline earth metal cation in the aqueous phase has a high concentration value that falls out of its maximum limits specified by the World Health Organization. Of the heavy metal ions, the concentrated Cu^2+^ and Ni^2+^ ions are perilous to human health, leading to diseases such as lung cancer, organ failure and nerve system destruction [7–9]. As a result, it has become an urgent issue to develop practicable, cost-effective methods for removing heavy metals from polluted waters.

Conventional techniques utilized to purify the contaminated water are chemical precipitation [10], ion-exchange [11], flotation [12], membrane filtration [13], electrochemical treatment [14], flocculation [15], and adsorption methods [16]. Among these purification techniques, adsorption is an overwhelmingly preferable recipe in that it is a facile, inexpensive and fast technique for removing heavy metal contaminants from aqueous media [17]. Adsorption falls into three different classes by depending on the textural properties of the solid adsorbent and the ambient parameters such as the temperature, concentration, pH value of the aqueous phase studied; physical-, chemical- and ion-exchange sorption. Each sorption type imposes different fiscal burdens on the regeneration step of the adsorbent. For instance, when it comes to chemical sorption, higher heat power is essential to break off chemical bonds between the adsorbate and adsorbent, leading to a higher purification cost [18]. In this context, the low investment including initial cost and land request make the adsorption method attractive more over the other techniques.

Commercial activated carbon (AC) is the most popular adsorbent that is often employed in the adsorption processes due to its high surface area. However, its high production cost made it important to find alternative adsorbents. Natural zeolites have been considered as an alternative candidate since their reserves are abundant in nature, particularly in Turkey [19]. Moreover, its mining and processing are easier and cheaper compared to its AC counterpart. Known as a type of clay, natural zeolites are microporous crystalline alumina silicates that are composed of three-dimensional networks of tetrahedron [AlO_4_]^5−^ and [SiO_4_]^4−^ molecules linked to together by mutual oxygen atoms. In the framework, the aluminum- and silicon-ions with a charge of 3^+^ and 4^+^, respectively, cause the inorganic structure to load with the negative charges; thereby the natural adsorbent exhibits higher affinity for heavy metal ions [20]. Natural zeolite includes Al, Si, Mg, Ca, K, Fe, Sr, Ti, Ba, and Zr at different mass ratios by its location. Previous studies based on the rheological properties of the zeolite uncovered that the natural zeolite comprises clinoptilolite (Na, K, Ca)_2–3_Al_3_(Al, Si)_2_Si_13_O_36_·12(H_2_O), quartz (SiO_2_), modernite ((Ca, Na_2_, K_2_) Al_2_Si_10_O_24_·7H_2_O), and feldspar (KAlSi_3_O_8_ −NaAlSi_3_O_8_ −CaAl_2_Si_2_O_8_) [21, 22]. Especially Na and Ca cations in the crystal structure of clinoptilolite can easily be replaced by inorganic and organic cations found as pollutants in domestic and industrial liquid wastes. In this context, it could be effectively utilized in the remediation of effluents formed by excess heavy metals during industrial processes.

The goal of this study is to examine the single and binary adsorption characteristics of Cu (II) and Ni (II) ions from an aqueous solution onto the zeolite. For this purpose, batch experiments were set in glass-beakers with the volume ranging from 50.0 mL to 1000.0 mL, investigated some parameters affecting on adsorption processes such as initial adsorbate concentration, pH, and temperature. Using the equilibrium concentrations of the Cu (II) and Ni (II) between adsorbate (called liquid phase) and adsorbent (called solid phase), adsorption equilibrium data were fitted into the Langmuir and Freundlich equations that were derived for both single and binary adsorption systems. Finally, some thermodynamic parameters such as the enthalpy change (ΔH), Gibbs free energy change (ΔG), and adsorption activation energy (Ea) were calculated to insight into the adsorption reaction mechanisms.

## 2. Materials and methods

### 2.1. Materials and chemicals

Natural zeolite, clinoptilolite (Na, K, Ca)_2–3_Al_3_(Al, Si)_2_Si_13_O_36_·12(H_2_O) employes as an adsorbent for the adsorption experiments, was naturally obtained from Yavu village, Sivas/Turkey. The natural zeolite was firstly grounded in a ball-mill jar with a stainless-steel (MM 400 Restch Miller) ball-to-powder mass ratio of 10:1, and then the powder sample was sieved with a mesh of 55 μm. The as-grinded powder samples were finally dried at 100 °C in a vacuum-oven for overnight. For the single sorption experiments, the appropriate amounts of CuSO_4_.5H_2_O and Ni(NO_3_)_2_.6H_2_O salts were separately dissolved in distilled water to prepare stock Cu (II) and Ni (II) solutions with the concentration of 1.00 mol/L. All batch adsorption experiments were performed in glass beakers of 250 mL. Artificial wastewater media was prepared using the stock solutions whose concentrations vary in the range of 100 to 1000 ppm. For binary sorption experiments, stock Cu (II) and Ni (II) solutions were mixed in stoichiometric amounts to prepare the artificial wastewater media in which the heavy metal-ion concentrations change in the range of 25 ppm to 1000 ppm. pH values of all the aqueous solutions were adjusted to be 5.0 using the NaOH and HCI of 1 mol/L.

### 2.2. Specific surface area

The specific surface area of the zeolite ((Na, K, Ca)_2–3_Al_3_(Al, Si)_2_Si_13_O_36_·12(H_2_O)) was determined using N_2_ adsorption-desorption technique (AUTOSORB 1C) at −190 °C. Before contacting wastewater, the powder adsorbent was evacuated until the pressure and temperature of the vacuum chamber arrive at a pressure of 60.1 Pa and the room temperature, respectively. Finally, it was heated up to 350 °C and evacuated again until a pressure of 1.3 Pa. This condition was sustained overnight.

### 2.3. Determination of moisture content in the adsorbent structure

To determine the amount of moisture in the adsorbent structure, 1000 g of zeolite was put into a porcelain crucible and the total weight was measured via a precision balance with 0.0001 g. This crucible was placed in a vacuum oven (WiseVen) and constantly heated at 65 °C for 24 h. The heated crucible was cooled to the room temperature in a desiccator to keep the solid sample away from the humidity available in the atmosphere. The crucible was weighed again. This process was repeated until there is a difference of 0.01 g between the initial weight and final weight of the crucible.

### 2.4. Adsorption studies

Single and binary adsorption experiments were conducted in a single-staged batch mode, using stoppered conical flasks (100 mL) on a magnetic stirrer. These tests were performed in the range of 25 to 35 °C, using 1000 g of the natural adsorbent. The free Cu (II) and Ni (II) concentrations in the aqueous medium were measured through a UV-Vis spectrophotometer (The Cary 60 UV-Vis spectrophotometer). By using the following equation derived from a mass balance between the solid and liquid phase, the amount of substance removed from effluent was calculated for each sorption study [23].


(Eq. 1) $$q=\frac{(C_{0}-C_{e})\times V}{m},$$

where C_0_ and C_e_ are the initial and residual concentrations of each metal contaminant in the solution at initial time and equilibrium, respectively (mg/L); V is the total volume of the liquid phase in which the remediation experiments of wastewater were carried out; m is the mass of used adsorbent (g). The adsorption efficiencies of Cu (II) and Ni (II) ions were calculated as follows:


(Eq. 2) $$\text{Sorption efficiency }(\%)=\frac{\left(C_{0}-C_{e}\right)}{C_{0}}\times 100.$$

In the second part of the adsorption experiments, the adsorption process of the mix of Cu (II) and Ni (II) ions was investigated at pH 5.0 which observed maximum sorption pH for both metal ions. The effect of metal ions concentration on the adsorption process was determined by keeping that either of both metal ions in aqueous solutions holds at stable concentration while another changed between 25.0 and 1000.0 mg/L. Binary adsorption experiments were also performed at different temperatures varying 25 and 35 °C.

The total yield of the binary adsorption experiments is the ratio of the equilibrium concentration of each metal ion trapped by zeolite to the initial total concentration of both metal ions.


(Eq. 3) $$\%\text{ad}=\frac{({\text{C}}_{(\text{ad},\text{d})\text{I}}+{\text{C}}_{(\text{ad},\text{d})\text{II}})/\text{X}}{\left({\text{C}}_{\text{0I}}+{\text{C}}_{\text{0II}}\right)},$$

where *C*_(_*_ad,d_*_)_*_I_* and C(ad,d)I *C*_(_*_ad,d_*_)_*_II_* are the concentration of the first and second metal ions remaining in the aqueous phase at equilibrium (mg metal ion / L solution), while *C*_0_*_I_* and *C*_0_*_II_* are the initial concentration of the first and second metal ions in aqueous medium (mg metal ion / L solution). X stands for the adsorbent mass per unit volume (g L^−1^).

### 2.5. The single adsorption isotherm equations

Adsorption equilibrium isotherms provide vital knowledge needed for a comprehensive understanding of an adsorption phenomenon. Langmuir’s and Freundlich’s isotherms were employed for present work to find out mathematically the correlation between the amount of the metal ion trapped by the sorbent and the amount of the metal contaminants in the aqueous solution [24, 25]. Langmuir isotherm is an equation that was theoretically derived making the assumptions that all active sites on the surface of adsorbent have the same adsorbate affinity and adjacent active sites are not affected from each other during the adhesion of the metal ions on the surface of Zeolite [26]. The linearized form of this isotherm model was represented as follows:


(Eq. 4) $$\frac{C_{e}}{q_{e}}=\frac{1}{K_{A}q_{m}}+\frac{1}{q_{m}}C_{e},$$

where C_e_ is concentration of metal ion remaining in aqueous phase at equilibrium (mg L^−1^), q_e_ is amount of adsorbate uptaked according to unit weight of adsorbent at equilibrium (mg g^−1^), q_m_ is the maximum amount of trapped adsorbate per unit weight of adsorbent at equilibrium (mg g^−1^), and K_A_ stands for equilibrium constant.

**Figure 1:**
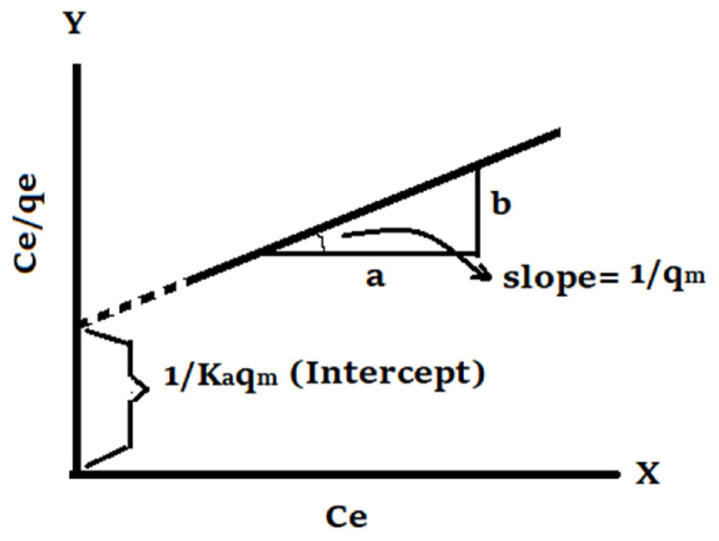
Representative graph of linearized Langmuir isotherm equation for single-adsorption systems.

The Langmuir isotherm constants “K_A_ and q_m_” could be estimated by plotting C_e_/q_e_ versus C_e_. The slope of the straight line in Figure 1 gives the value of 1/qm, and the interception point on the Y-axis in Figure 1 gives the value of 1/ K_A_q_m_.

The Freundlich sorption is an isotherm derived as a result of the empirical studies, and it is generally used to insight into the sorption event where multilayer sorption takes place on heterogeneous surfaces [27]. Its linearized form is expressed as follows:


(Eq. 5) $$\text{log}\;{\text{q}}_{\text{e}}={\text{logK}}_{\text{F}}+1/{\text{n logC}}_{\text{e}},$$

where C_e_ means the concentration of metal ion remaining in the aqueous phase at equilibrium (mg L^−1^), q_e_ is the amount of retained adsorbate per unit weight of adsorbent at equilibrium (mg g^−1^), K_F_ (mg g^−1^) and n are Freundlich isotherm constants.

**Figure 2:**
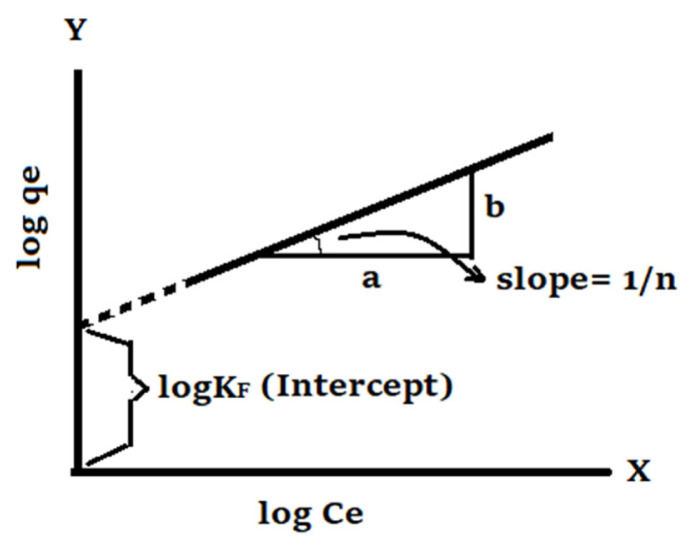
Representative graph of linearized Freundlich isotherm equation for single-adsorption systems.

K_F_ and n values could be found by plotting log q_e_ versus log Ce. The slope of the straight line in Figure 2 gives the value of 1/n, while the interception point on the Y-axis in Figure 2 gives the value of log K_F_.

### 2.6. The binary adsorption isotherm equations

Simplified forms of the Langmuir and the Freundlich sorption isotherms derived for multicomponent systems were used for the binary adsorption experiments. The modified Langmuir isotherm could be presented for the multicomponent systems as follows [28, 29]:


(Eq. 6) $${\text{q}}_{\text{denI}}=\frac{{{\text{Q}}_{\text{I}}}^{0}{\text{b}}_{\text{I}}\frac{{\text{C}}_{\text{denI}}}{\alpha }}{1+{\text{b}}_{\text{I}}\frac{{\text{C}}_{\text{denI}}}{\alpha }+{\text{b}}_{\text{II}}\frac{{\text{C}}_{\text{denII}}}{\beta }},$$

(Eq. 7) $${\text{q}}_{\text{denII}}=\frac{{{\text{Q}}_{\text{II}}}^{0}{\text{b}}_{\text{II}}\frac{{\text{C}}_{\text{denII}}}{\alpha }}{1+{\text{b}}_{\text{I}}\frac{{\text{C}}_{\text{denI}}}{\alpha }+{\text{b}}_{\text{II}}\frac{{\text{C}}_{\text{denII}}}{\beta }},$$

where q_denI_ and q_denII_ indicate uptake amount of each component onto adsorbent in simultaneous adsorption (mg/g), while C_denI_ and C_denII_ mean equilibrium concentration of first and second components in binary adsorption (mg/L), respectively. The coefficients “b_I_ and b_II_” are individual Langmuir adsorption isotherm constant of each component (mg/g) while the other coefficients “α and b” are the extended Langmuir isotherm constants for multicomponent adsorption systems.

The Freundlich isotherm for the binary adsorption system could be presented as [30, 31];


(Eq. 8) $${\text{q}}_{\text{denI}}=\frac{{\text{K}}_{\text{FI}}{\left(C_{denI}\right)}^{\left(n_{I}+x_{I}\right)}}{{\left(C_{denI}\right)}^{x_{I}}+y_{I}{\left(C_{denII}\right)}^{z_{I}}},$$

(Eq. 9) $${\text{q}}_{\text{denII}}=\frac{{\text{K}}_{\text{FII}}{\left(C_{denII}\right)}^{\left(n_{II}+x_{II}\right)}}{{\left(C_{denII}\right)}^{x_{II}}+y_{II}{\left(C_{denI}\right)}^{z_{II}}},$$

where q_denII_ and q_denII_ are the amount of each component hold onto adsorbent in simultaneous adsorption (mg/g) while C_denI_ and C_denII_ are equilibrium concentrations of first and second components in binary adsorption (mg/L), respectively. The constants “n_I_ and n_II_” are the individual Freundlich adsorption isotherm constant of each component. The coefficients “x_I_, x_II_, y_I_, y_II_, z_I_, z_II_ and K_FI_, K_FII_ are the extended Freundlich’s isotherm constants for multicomponent adsorption systems.

### 2.7. Thermodynamic parameters

The thermodynamic parameters of metal adsorption in the aqueous solution provide comprehensive information about the heavy metal uptake and the purification mechanism. Under the standard conditions, the change of enthalpy (ΔH°), entropy (ΔS°) and Gibbs free energy (ΔG°) values of the adsorption phenomenon are calculated as given in the following equation;


(Eq. 10) ΔG°=ΔH°-TΔS°.

The Gibbs free energy change (ΔG°), which ascribes whether the adsorption is spontaneous or not, could also be expressed in the following way;


(Eq. 11) ΔG°=-(RT ln Kc).

Kc is a distribution factor, expressing the ratio of the amounts of Cu (II) and Ni (II) ions in the solid phase to that of Cu (II) and Ni (II) in the liquid phase.

Substituting ΔG° value into this expression in terms of ΔH° and ΔS° ln Kc value, which is a function of temperature, could be expressed as follows:


(Eq. 12) $${lnK}_{C}=\frac{\left(\text{T}\Delta \text{S}{}^{\circ}-\Delta \text{H}{}^{\circ}\right)}{R}X\frac{1}{T}.$$

The standard enthalpy (ΔH°) and entropy (ΔS°) values are predicted from the intercept and slope of a plot between ln K_c_ against 1/T. ΔH° value ascribes whether the adsorption process was endothermic or exothermic.

## 3. Results and discussion

### 3.1 The Physicochemical parameters of the natural zeolite

**Figure 3:**
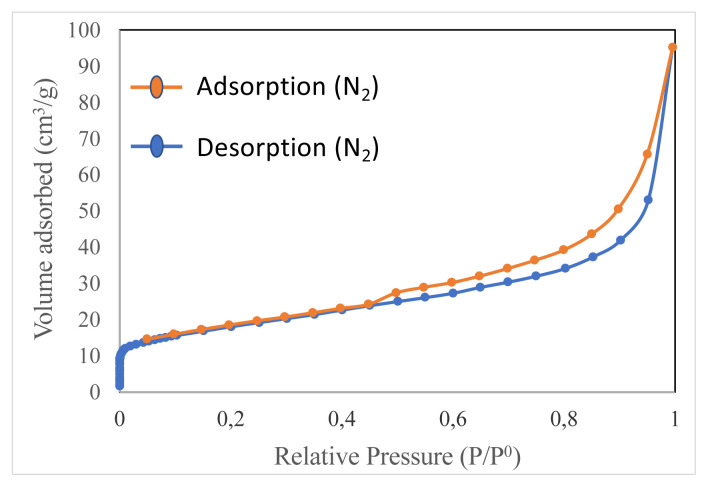
N_2_ adsorption-desorption isotherm curve of the natural zeolite obtained from Yavu village of Sivas city, Turkey.

The surface area is by far the most significant parameter when it comes to metal adsorption from wastewaters through natural adsorbents. Figure 3 indicates the BET isotherm curve for N_2_ adsorption/desorption, which is the typical S-shaped behavior of Type IV according to the IUPAC isotherm classification. This type of isotherm shows hysteresis that starts generally between the partial pressure values of 0.45–0.50 and continues until the values are between 0.95–0.98. The BET isotherm of the natural zeolite could be attributed to the presence of a vast number of larger mesopores, as well as micropores [32]. Multipoint BET results revealed the natural zeolite used has a surface area of 62.36 m^2^/g, which is compatible with the literature study [33, 34]. On the other hand, it was calculated that the solid sample contained 8% moisture by mass, which is due to the humidity in the air.

### 3.2. The single sorption studies

**Figure 4:**
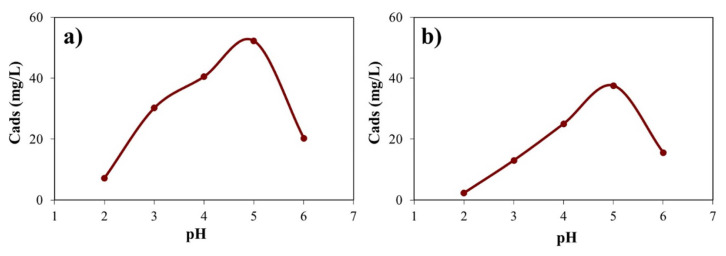
**(a)** The effect of the initial pH on the adsorption of Cu (II) by Zeolite (T: 20 °C; X: 1.00 g/L; Agitation Speed: 150 rpm), **(b)** The effect of the initial pH on the adsorption of Ni (II) by Zeolite (T: 20 °C; X: 1.0 g/L; Agitation Speed: 150 rpm).

The attachment of metal ions on the active points of the adsorbent gives rise to the formation of some spherical complexes. Since these complexes are formed through electrostatic interactions between metal cations and the active site with negatively charged, the pH of wastewaters plays a significant role in the removal of toxic heavy metals in effluents, affecting the surface morphology and behavior of the adsorbent during adsorption phenomena. This might be due to the fact that hydrogen ions in the aqueous phase compete with the positively charged metal ions to occupy the active sites of the solid adsorbent phase [35, 36]. As can be seen from Figures 4a and 4b, the adsorbed quantity of Cu (II) and Ni (II) on the zeolite increased in the strong acid value until the mild-acid. The maximum uptake of the metal ions was reached at pH = 5.0, which could ascribe to electrostatic repulsion and the saturation degree of the active sites on the adsorbent [37, 38]. The other parameters such as concentration and temperature affecting the adsorption process were evaluated at pH = 5.0.

**Figure 5:**
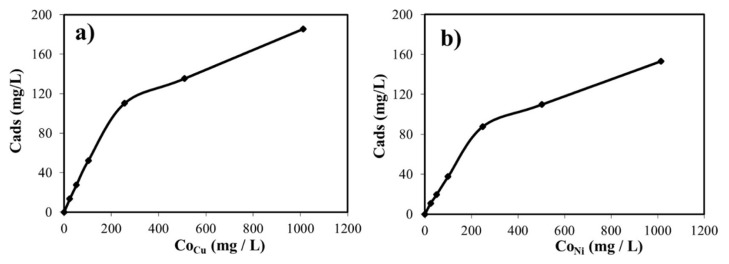
**(a)** Effect of the initial Cu (II) ion concentration on adsorption by zeolite (T: 20 °C; X: 1.00 g/L; Agitation Speed: 150 rpm), **(b)** Effect of the initial Ni (II) ion concentration on Adsorption by zeolite (T: 20 °C; X: 1.0 g/L; Agitation Speed: 150 rpm).

Artificial wastewater solutions were separately prepared for both Cu (II) ion and Ni (II) ion at different concentrations at constant pH = 5.0 to examine the metal uptake efficiency of the zeolite. Figures 5a and 5b indicate the relationship between the equilibrium concentration and the initial concentration of both metals. It is clearly seen that the sorption capacity of the adsorbent sharply increased up to the case in which 250 ppm initial metal concentrations of the aqueous phase. However, the uptake efficiency increased slightly above the mentioned value. It might be because the driving force to capture the heavy metal ions decreased due to the saturation degree of the adsorbent used [39, 40].

The effect of temperature on the sorption capacity was evaluated through the Langmuir and Freundlich isotherms which are employed for unary systems. Based on the outcomes of single-adsorption experiments performed at different temperatures, the isotherm constants calculated from the linear Freundlich and the Langmuir models were presented in Table 1. It shows that the ambient temperature leads the isotherm constants to increase under the mild-acid conditions. As a result, it might conclude that the metal uptake capacity and sorption reaction rate of the zeolite enhance with soaring temperature [41]. Moreover, Cu (II) removal efficiency of the zeolite is higher than its Ni (II) removal efficiency over all the temperatures, which indicates that the adsorbent gives a higher affinity to Cu (II) ion compared to its counterpart ion. These results are consistent with previously reported literature studies [42, 43].

**Figure 6:**
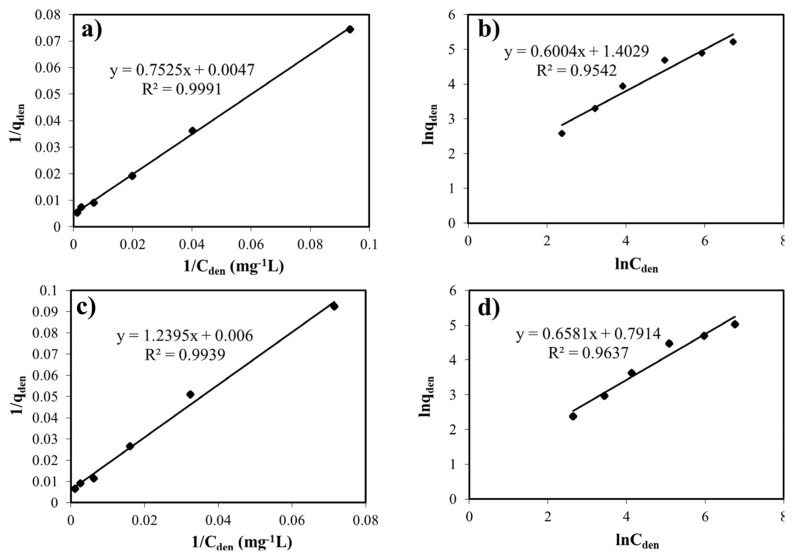
**(a)** The linearized Langmuir isotherm and **(b)** the linearized Freundlich isotherm model for the copper (II) uptake at pH 5.0 (T: 20 °C). **(c)** The linearized Langmuir isotherm and **(d)** the linearized Freundlich isotherm model for the nickel (II) uptake at pH 5.0 (T: 20 °C).

According to the experimental data, the Langmuir and Freundlich equations were fitted to the sorption isotherms and the squares of their regression (R^2^) are shown in Figure 6. The R^2^ values calculated for the Langmuir isotherm model are higher than those of the Freundlich isotherms, indicating that the equilibrium data for trapping the metal ions agree with the Langmuir sorption isotherm. In terms of the adsorption isotherm models, the results are compatible with the literature studies focusing on the metal sorption from wastewaters [44, 45]. That is, these results point out that the capture mechanism of the heavy metals on the zeolite relies on the physical process, as well as taking place single layer, homogeneous adsorption.

### 3.3. Binary adsorption systems

**Figure 7:**
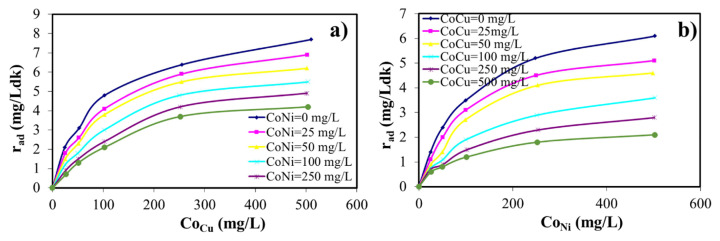
**(a)** Variation of the adsorption rate with time for the adsorption of Cu (II) on zeolite at different initial Ni (II) concentrations (T: 20 °C; X: 1.00 g/L; Agitation Speed: 150 rpm), **(b)** Variation of adsorption rate with time for the adsorption of Ni (II) on zeolite at different initial Cu (II) concentrations (T: 20 °C; X: 1.00 g/L; Agitation Speed: 150 rpm).

The binary metal sorption studies were performed at pH = 5.0 where Cu (II) and Ni (II) ions were trapped on the adsorbent with the maximum percentage. In binary metal aqueous phases, the initial Cu (II) concentration varies between 0 and 500 mg/L, while initial Ni (II) ion concentrations are 25, 50, 100, 250, and 500 mg/L. Figure 7 illustrates the relationship between the initial metal concentrations and the adsorption rates. Figure 7a shows the plot between the sorption kinetics of Cu (II) ion to the zeolite versus the different Ni (II) concentrations varying from 0 to 500 mg/L, keeping the Cu (II) concentrations constant. It was seen that the adsorbed amount and the adsorption rate of Cu (II) ion increased with the increasing of the concentration of Cu (II), but decreased with the increasing of the concentration of Ni (II). The results related to the adsorption rate of Cu (II) and Ni (II) were shown in Figures 7a and 7b.

To find out the effect of the initial metal concentration on the dual metal uptake system, the binary adsorption experiments were performed in an attempt to calculate adsorption yields by preparing the solutions including Cu (II) and Ni (II) at different concentrations combinations. Table 2 demonstrates that the more the total heavy metal concentration increase in the aqueous phase the less the adsorbent attracts binary metal ions to its own structure under the room temperature conditions. For the dual-metal ion system, the adsorbent “zeolite” delivered the best holding performance in case the initial concentrations of Cu (II) and Ni (II) ions were around 100 ppm and 25 ppm, respectively. The metal uptake results obtained from the binary adsorption systems are in the same range of the values in previous reports [43, 46].

**Figure 8:**
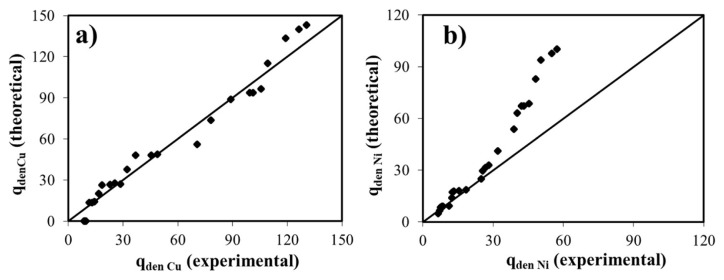
**(a)** Comparison of the experimental and the theoretical values of q_den_ in terms of the adsorption of Cu (II) on the line with 45**°** of slope, **(b)** Comparison of the experimental and the theoretical values of q_den_ in terms of the adsorption of Ni (II) on the line with 45**°** of slope.

Much as the sorption of binary-metal contaminants by zeolite has not been extensively examined up to date, this study extends the treatment to cover the antagonistic effects of both metal ions (Ni(II) and Cu(II) ) on the zeolite’s own structure. The simultaneous removal of the heavy metals by the zeolite was evaluated by the extended Langmuir and Freundlich isotherm models. The data collected from the adsorption experiment under the room temperature conditions and at pH = 5.0 were fitted into the competitive Langmuir isotherm model, and its constants were reported in Table 3. The constants of the mentioned isotherm model were predicted by using those of noncompetitive and competitive Langmuir isotherms derived for the removal of each metal ion at room temperature. The theoretical values of q_den_ were calculated by using these parameters and were compared with its empirical values in Figure 8. It was seen that the adsorption of the binary mixture of Cu (II) and Ni (II) agreed well with the Langmuir adsorption isotherm.

It was also examined whether the data acquired from the simultaneous dual-adsorption experiments were fitted to the Freundlich isotherm. The Freundlich adsorption constants were calculated by using K_F_ and 1/n which were predicted for the single metal adsorption. The theoretical q_den_ and % error values were calculated by using the constants of the Freundlich adsorption model derived for the single and binary adsorption phenomena, and the results were listed in Table 3 to compare with the experimental q_den_ values. The percent magnitude of the error for the competitive metal uptake and the relationships between predicted and experimental outcomes for each model are acceptable at low initial mixture concentrations; however, the correlation deviated considerably in the range of high mixture concentrations. Considering % error values calculated for the extended Langmuir and Freundlich isotherm models, Figure 8 also indicates that the former sorption model provides the best correlation between the experimental and theoretical adsorption data for copper (II) metal ion at room temperature and at pH = 5.0 compared to its counterpart ion. To sum up, it could be concluded that the competitive Langmuir model provides a more realistic definition of the multiadsorption phenomena at the studied concentration range. From multicomponent adsorption isotherms of view, the binary sorption results are in good agreement with the previous literature reports [43, 47].

### 3.4. The thermodynamic characterization of the adsorption by the natural zeolite

During the experiments conducted for the uptake of Cu (II) and Ni (II) on zeolite at temperatures ranging from 20 to 35 °C, the enthalpy changes (ΔH) were calculated from the slope of the linear plot of ΔG versus T. The results were listed in Table 4. The enthalpy values were found to be positive for each metal ion, indicating that the sorption reactions of each metal ion were endothermic. This result is similar to those of other clay materials in the literature [48].

Thanks to the activation energy which is the minimum amount of energy to take place the metal uptake reaction in the interface between adsorbent and aqueous phase, the kind of adsorption could be determined for the uptake of both metal ions [49]. Using the Arrhenius equation arranged as a linear equation with the form lnk = −EaRT + lnA, the activation energy for the metal adsorption reaction was calculated by finding the slope of the line. The Ea values for the adsorption of each metal contaminant were found to be 33.57 and 41.33 kJ/mol, respectively, suggesting that the sorption of Cu (II) and Ni (II) contaminants on Zeolite involves both chemical adsorption and physical adsorption [50, 51]. Gibbs’s free energy changes (ΔG) calculated for the adsorption of two heavy metals on the zeolite were listed in Table 5. For the Cu (II) uptake, ΔG values are negative between 25 ppm and 100 ppm, which means that the adsorption reaction mechanism for Cu (II) contaminant is exothermic in the aforementioned concentration range. This result shows that Cu (II) metal uptake by the zeolite is spontaneous and thermodynamically favorable in the low concentration ranges. Above 100 ppm of the solution concentration, ΔG values, however, were predicted to be positive, referring to endothermic reaction mechanism. This might be because the rate of metal contaminants per active site on the adsorbent increased in the high concentration ranges [52]. For the Ni (II) uptake, all ΔG values were found to be positive in all the concentration values except for the 25 and 50 ppm at 35 °C, indicating that the Ni (II) adsorption mechanism is carried out as endothermic. Considering each metal uptake, it could be concluded that the adsorbent zeolite has a higher affinity for Cu (II) contaminants than the other ion, which supports the results of single and binary metal adsorption experiments.

## 4. Conclusion

For this study, the natural zeolite was unearthed from the Yavu region of Sivas/Turkey and employed as an adsorbent for the competitive and noncompetitive metal adsorption by setting up batch wastewater systems. The single metal sorption performance of the natural adsorbent was optimized by adjusting the solution pH, initial metal concentration, and the ambient temperature. For both Cu (II) and Ni (II) adsorption, the best solution pH value was found to be around 5.0 in the monocomponent sorption experiment at the room temperature condition. The maximum sorption capacity of the natural zeolite was found to be 250 ppm for the remediation of each metal ion from the synthetic wastewater with pH = 5.0. The data obtained from the individual metal uptake experiments were applied to the Langmuir and Freundlich isotherm models which were derived for the single adsorption phenomena. Within the concentration ranges of heavy metals pertaining to this study, from 25 to 1000 ppm, the individual metal uptake is satisfactorily described by the Langmuir isotherm model. The affinity order of the natural zeolite was Cu^2+^ > Ni^2+^ in noncompetitive adsorption systems, which is compatible with the literature studies [43].

The simultaneous adsorption of Cu (II) and Ni (II) contaminants on the zeolite was executed at pH = 5.0 in the batch glass reactor systems. The natural zeolite held the greatest removal potential in a mixture where the initial concentrations Cu (II) and Ni (II) were 100 ppm and 25 ppm, respectively. The adsorption results in multicomponent systems demonstrate that the combined effects of the metallic effluent on the natural zeolite are antagonistic. In other words, the ability of the adsorbent to bind simultaneously Cu (II) and Ni (II) in solution decreased with increasing the initial concentration of each metal ion in the aqueous phase. The equilibrium metal uptake data were applied to the competitive Langmuir and Freundlich isotherm equations to describe the binary adsorption equilibria in the batch systems. It has been found that the competitive Langmuir and Freundlich isotherm models procured the best fit to the experimental data in the low range of competing metal ions.

The activation energies of Cu (II) and Ni (II) sorption (Ea) were calculated as 33.57 and 41.33 kJ/mol. The high Ea values demonstrate that the noncompetitive metal uptake process by the natural zeolite may be physical and chemical adsorption. For the Cu (II) removal process, the Gibbs free energy (ΔG°) values were found negative for Cu (II) adsorption in the low concentration values, from 25 ppm to 100 ppm, confirming that the copper (II) uptake is spontaneous and applicable in the mentioned concentration range. However, above 100 ppm, The ΔG° values were found positive, indicating that the metal uptake process is endothermic. As for Ni (II) adsorption, ΔG° values were found positive in the concentration range varying between 25 ppm and 1000 ppm. The positive ΔG° values suggest that the Ni (II) uptake by the natural zeolite obtained from Yavu village is not spontaneous. These results elicit that the natural zeolite showed a stronger affinity toward Cu (II) ion than Ni (II). The ΔH° values are positive for each metal ion, which means that the removal process of the divalent metal cations is endothermic at room temperature.

This study could enable the estimate of the noncompetitive and competitive adsorption equilibria of the Cu (II) and Ni (II) metallic effluents by the natural adsorbent to extrapolate if empirical data are not available for a certain level concentrations of the singular and binary metal.

## Figures and Tables

**Table 1:** The Langmuir and Freundlich constants obtained from the isotherms in the adsorption of Cu (II) and Ni (II) at different temperatures.

Adsorption data for the Cu (II) ion				
Temperature (°C)	Langmuir		Freundlich	
	Q° (mg adsorbate/g adsorbent)	b (L/mg)	K_F_	n
20	212.766	0.00625	4.06698	1.66556
25	227.273	0.00631	4.19377	1.63339
30	250	0.0064	4.58367	1.62022
35	270.27	0.00805	6.12207	1.68976
**Adsorption data for the Ni (II) ion**				
**Temperature (**°**C)**	**Langmuir**		**Freundlich**	
	**Q**° **(mg adsorbate/g adsorbent)**	**b (L/mg)**	**K** **_F_**	**n**
20	166.667	0.00484	2.20648	1.51953
25	172.414	0.00511	2.26551	1.48987
30	188.679	0.00564	2.77153	1.51745
35	232.558	0.00596	3.88297	1.59898

**Table 2:** % Total adsorption of Cu-Ni in the binary adsorption system.

Co_Cu (mg/L)_	Co_Ni (mg/L)_	% Top ads	Co_Cu (mg/L)_	Co_Ni (mg/L)_	% Top ads	Co_Cu (mg/L)_	Co_Ni (mg/L)_	% Top ads
24.1	0	55.6016598	25.3	24.9	43.0278884	24.8	50.7	35.761589
52.3	0	52.581262	52.2	25.5	43.2432432	50.7	49.8	35.820896
102.4	0	50.9765625	102.3	25.3	44.1222571	102.3	51.2	37.850163
255.6	0	43.114241	254.3	24.7	40.3942652	254.6	51.3	37.136319
510.1	0	26.5438149	502.8	25.9	25.9126159	501.8	49.8	24.927484
**Co** **_Cu (mg/L)_**	**Co** **_Ni (mg/L)_**	**% Top ads**	**Co** **_Cu (mg/L)_**	**Co** **_Ni (mg/L)_**	**% Top ads**	**Co** **_Cu (mg/L)_**	**Co** **_Ni (mg/L)_**	**% Top ads**
24.8	99.8	30.3772071	25.8	249.8	19.7931785	26.4	502.1	12.450331
49.7	98.8	30.4444444	50.8	253.2	19.6381579	50.8	498.7	12.647862
101.7	99.6	30.9488326	102.3	251.8	20.4462016	102.3	503.1	13.032706
249.9	102.3	32.3395798	252.2	253.8	23.1225296	252.2	502.1	15.736444
503.7	101.2	22.7640932	501.9	252.2	18.7243071	503.8	503.1	14.043103

**Table 3:** The Langmuir and Freundlich adsorption constants derived for the binary mixture of Cu (II) and Ni (II) and % error values.

Langmuir constants	Copper (II)	Nickel (II)
**Q° (mg adsorbate/g adsorbent)**	212.766	166.6667
**b (L/mg)**	0.006246	0.004841
**α**	1.03586	1.338194
**b**	1.05124	1.720407
**% error**	0.895451	1.334985
**Freundlich constants**	**Copper (II)**	**Nickel (II)**
**x**	0.559869	0.692643
**y**	0.443928	0.727683
**z**	0.572842	0.772552
**% error**	14.77326	59.90584

**Table 4:** The enthalpy changes at the adsorption of Cu (II) and Ni (II) by zeolite.

Zeolite	ΔH (kj mol^−^^1^)
**Copper (II)**	15.05
**Nickel (II)**	10.88

**Table 5:** Thermodynamic parameters for the adsorption of Cu (II) and Ni (II) by the zeolite.

Thermodynamic values for the adsorption of Cu (II) by zeolite						
T (°C)	25 ppm		50 ppm		100 ppm	
	Kc	ΔG (j/mol)	Kc	ΔG (j/mol)	Kc	ΔG (j/mol)
**20**	1.2523	−548.12716	1.10887	−251.74217	1.0398	−95.168431
**25**	1.3614	−764.38279	1.16937	−387.65723	1.1061	−249.88942
**30**	1.5216	−1057.4077	1.31417	−688.24932	1.2277	−516.73912
**35**	2.0654	−1857.2842	1.80953	−1518.6801	1.7254	−1396.7497
**T (°C)**	250 ppm		500 ppm		1000 ppm	
	Kc	ΔG (j/mol)	Kc	ΔG (j/mol)	Kc	ΔG (j/mol)
**20**	0.7579	675.239459	0.36136	2479.58779	0.2248	3636.0684
**25**	0.8995	262.475143	0.40512	2238.66061	0.2505	3429.2743
**30**	1.0779	−189.01082	0.47351	1883.24187	0.2883	3133.6062
**35**	1.3336	−737.17284	0.54794	1540.50603	0.3285	2850.6324
**Thermodynamic values for the adsorption of Ni (II) by zeolite**						
**T (°C)**	25 ppm		50 ppm		100ppm	
	Kc	ΔG (j/mol)	Kc	ΔG (j/mol)	Kc	ΔG (j/mol)
**20**	0.7725	628.773871	0.6348	1107.1456	0.603	1232.090924
**25**	0.84389	420.515819	0.6872	929.54518	0.6221	1175.936011
**30**	1.02083	−51.925801	0.805	546.36093	0.7662	670.8918725
**35**	1.30565	−682.94218	1.2622	−596.334	0.9511	128.2726235
**T (°C)**	250 ppm		500 ppm		1000 ppm	
	Kc	ΔG (j/mol)	Kc	ΔG (j/mol)	Kc	ΔG (j/mol)
**20**	0.5452	1477.67768	0.2791	3108.4771	0.1778	4207.174266
**25**	0.61046	1222.79154	0.3168	2848.0601	0.2012	3972.85976
**30**	0.72766	800.884927	0.3596	2576.349	0.228	3724.38702
**35**	0.86364	375.39308	0.4104	2280.8919	0.2561	3488.554441
